# Myeloperoxidase gene-463G > A polymorphism and premature coronary artery disease

**DOI:** 10.1590/S1415-47572009005000035

**Published:** 2009-06-01

**Authors:** Chen Zhong, Yin Quanzhong, Ma Genshan, Zhang Hua, Zheng Ruolong, Wang Jiahong, Gao Chunheng

**Affiliations:** 1 Department of Cardiology, The Affiliated ZhongDa Hospital of Southeast University, NanjingP.R. China; 2 Department of Cardiology, The Affiliated JiangYin Hospital of Southeast University, JiangyinP.R. China

**Keywords:** premature coronary artery disease, myeloperoxidase, protective genetic polymorphism

## Abstract

We investigated the association between myeloperoxidase gene -463G > A polymorphism and premature coronary artery disease (CAD) in two Chinese population samples: 229 patients and 230 controls. Genotypes were determined by ligase detection reaction-polymerase chain reaction sequencing and the grouping technique. We found lower frequencies of both the A/A genotype and the A allele in patients (p < 0.05). Multivariate logistic regression showed that the risk of premature CAD in subjects carrying the AA genotype was reduced by 83% in relation to individuals carrying the G/G genotype (OR = 0.172, 95% CI: 0.057-0.526, p = 0.002). Our results indicate that -463G > A polymorphism of the myeloperoxidase gene is associated with premature CAD in Chinese individuals, suggesting that the AA genotype is a protective factor against premature CAD.

Myeloperoxidase (MPO) is a peroxidase enzyme produced by white blood cells (mainly neutrophil granulocytes and mononuclear cells). It represents a class of hemeproteins belonging to the heme peroxidase family. Several single nucleotide polymorphisms (SNPs) are present at sp1 binding sites in the promoter region of the *MPO* gene, including variants -463G/A, -129G/A, -V53F, -A332V, -638C/A, I642L, and IVS11-2A/C. To date, several studies have shown that the -463G/A, -129G/A, -V53F, -A332V, and -638C/A SNPs and MPO levels are risk factors in coronary artery disease (CAD) (Nikpoor *et al.*, 2001; Brennan *et al.*, 2003; Chevrier *et al.*, 2003, 2006; Rutgers *et al.*, 2003; Nicholls and Hazen, 2005; Stefanescu *et al.*, 2008). Although *MPO* gene -463G > A polymorphism was first proven to be associated with CAD in a study by Nikpoor *et al.*, (2001), the inclusion criteria used were disputable, since coronary stenosis of ≥ 30% qualified this as being a CAD case. More importantly, genetic factors for CAD may be easier to detect in younger than in older groups, for in the latter many coexisting environmental factors might contribute to the progress of CAD. Studies conducted with younger CAD patients would thus help to reveal genetic factors relevant to CAD, if existent. Furthermore, to date no reports have dealt with the association between *MPO* gene -463G > A polymorphism and premature CAD. This was the aim of the present study.

From October, 2005 to May, 2008, 459 patients scheduled to undergo coronary angiography (CAG) for chest discomfort or suspected CAD, were enrolled in this study. 229 patients (men aged 36-54 years and women aged 39-64 years) with documented premature CAD (Kareinen *et al.*, 2001) constituted the case group. CAD was considered to be premature if occurring in men with less than 55 years of age and women with less than 65 years at the time of diagnosis. Patients with CAD presented stenosis (≥ 50%) in at least one main coronary artery or had undergone myocardial infarction defined according to WHO criteria (WHO, 1990). 230 age- and sex-matched patients without detectable coronary stenosis in CAG were used as controls. All patients presenting infectious diseases, valvular and congenital heart disease, the X syndrome, connective tissue disease, rheumatism, malignant tumor, trauma, severe liver or kidney disease, or non-coronary artery thrombotic disease, or who were making use of anti-inflammatory drugs, were excluded from the study.

Anthropometric measurements and blood pressure determination were performed according to standard protocols. Body mass index (BMI) was calculated as the weight in kilograms divided by the square of the height in meters. Information on smoking habits and family history was obtained from all sampled individuals. The study protocol was approved by the Ethics Committee of the affiliated Zhong Da Hospital of Southeast University, all participants giving prior informed consent.

Venous blood was drawn from fasting subjects with 0.1% EDTA. One sample from each subject was used to analyze fasting blood sugar content (FBS), total cholesterol (TC), triglycerides (TG), LDL-cholesterol, HDL-cholesterol (HDL-C), apolipoprotein A1 (apoA1), apolipoprotein B (apoB) and lipoprotein a [Lp(a)]. A further sample was centrifuged (3,000 rpm for 15 min), the blood cells then being separated and stored in micro-tubes -70 °C for genomic DNA extraction. Plasma concentrations of TC, TG, HDL-C, and LDL-C were determined by standard biochemical methods through using a chemistry analyzer (Beckman Coulter SYNCHRON LX20 Clinical System, Fullerton, CA, USA).

DNA was obtained from blood cells by means of a whole-blood genome DNA extraction reagent kit [Axygene Biotechnology (Hangzhou) Limited (Hangzhou City, China)], according to manufacturer's instructions.

Genotyping was performed through ligase detection reaction (LDR) (Favis *et al.*., 2000; Xiao *et al.*, 2006) and polymerase-chain reaction (PCR) sequencing-type technology. Primer sequence included the use of upstream (5'-CGG TAT AGG CAC ACA ATG GTG AG-3') and downstream (5'-GCA ATG GTT CAA GCG ATT CTT C-3') primers. The PCR product was a 350 bp fragment including -463G > A SNP. Probe sequences used were P-G AAGAATCGCTTGAACCATTGCACATTTTTTTTTTTTTTTT – FAM, TTTTTTTTTTTTTTTCCCAGCTACTC GGGAGGCTCAGGCG,TTTTTTTTTTTTTTTTTTTCCCAGCTACTCGGGAGGCTCAGGCA for rs2333227 SNP modified, rs2333227_C and rs2333227_T, respectively. A Tris-EDTA loading buffer was prepared according to the molecular weight of the respective primer and fluorescent probe, and diluted to 50 pmol/μL as the stock solution. Thermal cycling of the PCR process was performed under the following conditions: 70 °C for 15 min, followed by 35 cycles of 94 °C for 30 s, 60 °C for 90 s, 72 °C for 1 min and a final extension of 72 °C for 10 min. PCR products were visualized after electrophoresis on 3% agarose gel. LDR was carried out under the following conditions: 95 °C for 2 min, followed by 35 cycles of 94 °C for 30 s and 60 °C for 2 min. The sequence was detected and genotypes were obtained through a sequencing system (ABI377, Application Biotechnology Corporation).

Statistical analyses were undertaken using SPSS 12.0 software and included descriptive statistics, Student's t-tests, comparisons of gene and genotype frequencies between the two groups, regression and multivariate analyses, and the calculation of odd-ratios with corresponding 95% confidence intervals.

The analysis of baseline characteristics, lipids, and glucose levels in patients with premature CAD and in the control group is shown in Table 1. The prevalence of smoking (44.1% *vs.* 23.5%), hypertension (64.2% *vs.* 51.7%), diabetes (22.3% *vs.* 11.7%), and a family history of CAD (36.7% *vs.* 21.7%), were significantly higher among patients than in the control group. The levels of FBS, TC, TG, LDL-C, apoB, and Lp(a) in the case group were significantly higher than those in the control group. No significant differences were detected in the levels of HDL-C and apoA1, as well as in preference by sex, average age and body mass indexes between cases and controls.

The polymorphism frequency distribution between the case and control groups is shown in Table 2*.* The GG genotype was found to be the most frequent genotype in both cases (66.4%) and controls (58.7%). Frequencies of the AA genotype and the A allele were significantly lower among patients than controls.

With respect to relationship between risk factors, MPO gene -463G > A polymorphism and premature CAD (Table 3), no significant differences were detected for BMI, FBS, all lipid parameters, hypertension, smoking, diabetes and a family history of CAD, when the genotypes of cases and controls were compared. Among all the covariates examined as potential confounders, only TC, diabetes mellitus, smoking and a family history of CAD contributed to the final multivariate model. The other variables, including BMI, FBS, TG, apolipoproteins, and hypertension, were not identified as independent variables. Adjusting to these covariates unmasked a significant association between *MPO* gene -463G > A genotypes and premature CAD. In patients carrying the AA genotype, the risk of premature CAD was reduced by 83% in relation to that of individuals carrying the G/G genotype (OR = 0.172, 95% CI: 0.057~0.526, p = 0.002).

We detected a significantly decreased risk of premature CAD in those individuals among native Chinese bearing the *MPO* gene -463 AA genotype. The *MPO* gene is located in chromosome 17q23-q24, and its expression is regulated by nutrilites (Denzler *et al.*, 1997). The *MPO* -463G > A polymorphism, located at the promoter region, was first detected by Austin *et al.* (1993) in a study on acute myeloid leukemia patients. More recently, this same gene polymorphism was proven to be a risk of both CAD (Nikpoor *et al.*, 2001) and cardiovascular events in CAD patients, the A allele being associated with less probability of developing cardiovascular disease (Asselbergs *et al.*, 2004). The patients used in these studies were significantly older and the angiography criteria for CAD cases were disputable due to coronary stenosis of ≥ 30% being qualified as a CAD case (Nikpoor *et al.*, 2001). Since genetic factors for CAD may be easier to detect in younger groups than in older ones, we assumed that this gene could be closely related with premature CAD. Indeed, by using groups of younger subjects in our case-control study, we found that the risk of premature CAD was significantly reduced in Chinese patients with the AA genotype, in accordance with the results obtained for CAD by Nikpoor *et al.* (2001) when using older subjects.

It has been reported that *MPO* gene -463G > A polymorphism is related to changes in lipid levels (Nikpoor *et al.*, 2001), and is a mechanism that may be involved in the oxidation of low-density lipoprotein, the high levels of MPO increasing the brittleness of artery plaques, thereby converting the plaque from the stable to the unstable state, thus increasing the risk of acute coronary syndrome (Sugiyama *et al.*., 2001; Ndrepepa *et al.*, 2008). The *MPO* -463A allele could interfere with the binding sites of the sp1 transcription factor, by reducing the level of *MPO* gene expression in its role in atherosclerotic plaque formation, thus having a definite impact on the risk of CAD. Meanwhile, MPO could promote the oxidation of HDL-C and affect the reverse transport role of cholesterol, thereby interfering in the development of atherosclerosis. The multivariate logistic regression that we performed showed that TC, diabetes mellitus, smoking and a family history of CAD are independent predictors of premature CAD, thus confirming that these are really traditional cardiovascular risk factors contributing independently to this disease (Schaefer *et al.*, 1994; Bostom *et al.*, 1996).

Our study and the results thence naturally present certain limitations, since the study-group was obtained from a single hospital in Southeast China, and the samples might not include all the characteristics of patients from other centers. It is also generally accepted that CAD is a disease produced by both multiple genes and environmental factors, and many other genes could be potential candidates for this sickness. Furthermore, since the *MPO* AA genotype is relatively infrequent, the actual value of genotype detection in the primary prevention of CAD may be disputable.

## Figures and Tables

**Table 1:** Comparison of baseline characteristics between CAD case and control groups (mean±SD)

	Controls (n = 230)	Cases (n = 229)
Age (ys)	52.90 ± 7.42	52.33 ± 6.97
Sex, male (%)	125 (54.3)	131 (57.2)
BMI (kg/m^2^)	24.56 ± 3.27	25.10 ± 2.90
FBS (mmol/L)	5.56 ± 1.38	6.07 ± 2.12^*^
TC (mmol/L)	4.24 ± 0.98	4.74 ± 1.06^*^
TG (mmol/L)	1.97 [0.66, 7.14]	2.18 [0.72, 6.64]^*^
HDL-C (mmol/L)	1.15 ± 0.27	1.13 ± 0.28
LDL-C (mmol/L)	2.57 ± 0.73	2.88 ± 0.72^*^
apoA1 (mmol/L)	1.26 ± 0.22	1.21 ± 0.28
apoB (mmol/L)	0.82 ± 0.30	0.94 ± 0.28^*^
Lp(a) (mg/L)	153.80 ± 220.69	266.39 ± 303.51^*^

Values are expressed as mean±SD; *, p < 0.01 (cases compared to controls). BMI, body mass index; CAD, coronary artery disease; FBS, fasting blood sugar; HDL-C, high-density lipoprotein-cholesterol; TC, total cholesterol; TG, triglyceride; LDL-C, low-density lipoprotein-cholesterol; apoA, apolipoprotein A; apoB, apolipoprotein B; Lp(a), lipoprotein (a).

**Table 2:** Genotype and allele distribution in CAD cases and controls.

	Controls (n/%)	CAD cases (n/%)	OR (95%CI)
Total			
GG	135/58.7	152/66.4	
AG	74/32.2	69/30.1	0.339 (0.145~0.790)*
AA	21/9.1	8/3.5	0.409 (0.170~0.984)*
p	0.030		

Relative allele frequencies			
Allele G	344/74.8	373/81.4	
Allele A	116/25.2	85/18.6	0.676 (0.493~0.927)*
p	0.015		

p, the significance level of comparison between cases and controls (χ^2^ test); *CAD,* coronary artery disease; OR, odds ratio; 95% CI, 95% confidence interval; *, a significant (p < 0.05) test value.

**Table 3:** Baseline risk factors and genotypes related to the risk of premature CAD after multivariate logistic regression analysis.

Factors	B	P	OR	95% CI
Genotypes				
GG		0.008	1*	
AA	-1.758	0.002	0.172	0.057~0.526
TC	0.838	0.008	2.365	1.390~3.644
Diabetes mellitus	1.130	0.007	3.102	1.368~7.010
Smoking	1.589	0.000	4.899	2.384~10.067
Family history of CAD	0.824	0.013	2.279	1.194~4.351

B, partial regression coefficient; CAD, coronary artery disease; CI, confidence interval; OR, odds ratio; TC, total cholesterol. * = Reference.
